# Clinical application of oral meglumine diatrizoate esophagogram in screening esophageal fistula during radiotherapy for esophageal cancer

**DOI:** 10.1097/MD.0000000000010668

**Published:** 2018-05-04

**Authors:** Lidan Geng, Rong Wu, He Hu, Yu Zhao, Lingli Fan, Zhenhua Zhao, Dongbiao Liao, Musheng Li, Miao Xiang, Ying Ma, Xiaobo Du

**Affiliations:** a Department of Oncology, Mianyang Central Hospital, Mianyang; b Department of Oncology, Affiliated Hospital of North Sichuan Medical College, Nan Chong; c Department of Oncology, Yan Ting County Cancer Hospital, Yan Ting; d Department of Oncology; e Department of Radiology; f Department of Scientific Research and Education, Mianyang Central Hospital, Mianyang, People's Republic of China.

**Keywords:** chemotherapy, complication, esophageal cancer, esophageal fistula, meglumine diatrizoate, radiotherapy

## Abstract

**Introduction:**

Esophageal fistula is a serious and common complication of radiotherapy for esophageal cancer. Therefore, early diagnosis and treatment is necessary. Because of side effect of barium esophagography, it cannot be used to screening esophageal fistula during radiotherapy. Meglumine diatrizoate is an ionic contrast agent, its adverse reactions were rarely seen when it was used in the body cavity. The purpose of this trial is identified the sensitivity and specificity of oral meglumine diatrizoate in an esophagogram for screening esophageal fistula during radiotherapy.

**Methods/design:**

This trial was a prospective, multicenter, diagnostic clinical trial. A total of 105 patients with esophageal cancer will swallowed meglumine diatrizoate and underwent a radiographic examination weekly during radiotherapy, medical personnel observed the esophageal lesions to determine whether an esophageal fistula formed. If an esophageal fistula was observed, esophagofiberoscopy and/or computer tomography was used to further confirm the diagnosis. And the sensitivity and specificity of meglumine diatrizoate should be calculated for screening esophageal fistula during radiotherapy.

**Discussion:**

To our knowledge, this study protocol is the first to identify the sensitivity and specificity of oral meglumine diatrizoate in an esophagogram for screening esophageal fistula during radiotherapy. If oral meglumine diatrizoate can be used to screening esophageal fistula, more patients will benefit from early detection and treatment.

## 1 Introduction

Esophageal cancer, one of the common clinical tumors, is highly malignant and has a poor prognosis.^[1]^ Esophageal cancer is an enormous burden in China. A distinctive feature of esophageal cancer in China is its uneven burden between rural and urban areas. The rates of esophageal cancer are 2- to 10-fold higher in rural areas compared with urban areas.^[2]^ High-risk areas throughout China have been defined based on previous national mortality surveys.^[3]^ Some high-risk areas include regions surrounding the Taihang Mountains in North Central China and Yanting, in northeastern Sichuan Province.^[4]^ Surgery is the primary treatment modality for esophageal cancer. However, surgical treatment of advanced esophageal cancer presents difficulties in resection, postoperative complications, and high mortality, so chemoradiotherapy is preferable.^[5–9]^

Chemoradiotherapy can induce fistula formation by damaging the walls of the esophagus and adjacent organs.^[10,11]^ During radiotherapy, severe complications such as tracheoesophageal fistula, esophageal perforation, esophageal stricture, fatal arterial hemorrhage, and pericardial effusion may occur.^[12]^ Esophageal fistula is a serious and common complication of radiation therapy. Locally advanced esophageal carcinoma can be complicated by fistulae in about 5% to 13% of cases.^[13–16]^ The treatment-related (esophageal brachytherapy and chemotherapy) esophageal fistula rate is 14%.^[17]^ Because of the potentially high mortality associated with these complications,^[18–20]^ early diagnosis and treatment is important.^[21]^ Clinically, esophageal fistulae must be suspected on the basis of a history of vomiting, chest pain, fever, and subcutaneous emphysema.^[22,23]^ If patients exhibit no symptoms, they do not undergo screening tests until large fistulae have formed. So, the screening of esophageal fistula is necessary during radiotherapy.

Esophagofiberoscope can be used in patients with esophageal fistula. But it is an invasive operation, weekly inspection can lead to esophageal mucosal lesions or injury; in addition, the laboratory fee and risk are large. It is not suitable for follow-up screening of esophageal fistula during radiotherapy. Barium esophagography has been widely used in the screening and diagnosis of esophageal cancer, but it is not suitable for screening esophageal fistula. Barium esophagography offers some benefit, the risk of complications is high; complications include inflammation of surrounding tissue, aspiration of contrast agent and death, and cost of examination.^[24–30]^ When esophageal perforation is clinically suspected, the examination should initially be performed with water-soluble contrast agents such as meglumine diatrizoate, which is rapidly absorbed from the mediastinum.^[24]^

Meglumine diatrizoate, an ionic contrast agent, is a colorless or faint yellow liquid. It has a short half-life and is rapidly metabolized in the kidneys. Patients rarely experience adverse reactions, and its price is lower than other iodine contrast agents. Therefore, meglumine diatrizoate esophagogram seems best suited for screening esophageal fistula during radiotherapy. However, the sensitivity and specificity of diagnosis of esophageal fistula have not been reported.

We designed a study to identify the sensitivity and specificity of oral meglumine diatrizoate in an esophagogram for screening esophageal fistula and gain a clearer understanding of its diagnostic value for esophageal fistula. We also want to understand patient prognosis after early detection and timely treatment of esophageal fistula during radiotherapy, in order to select the best early screening examination.

## 2 Objectives

### 2.1 Primary

The primary outcome is the sensitivity and specificity of oral meglumine diatrizoate esophagogram for screening esophageal fistula during radiotherapy.

### 2.2 Secondary

The secondary outcome is the healing rate of esophageal fistula, which is early detected and treated.

## 3 Methods/design

### 3.1 Recruitment and study design

**Figure 1:**
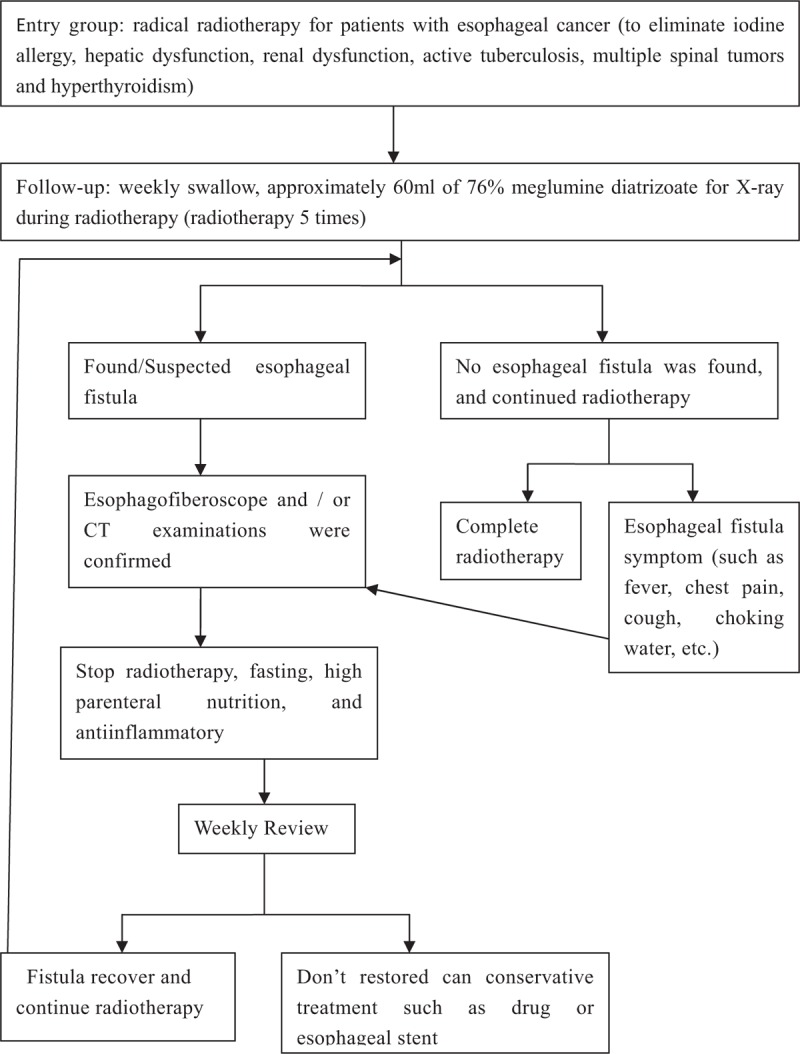
Overview of the diagnostic pathway for esophageal fistula by oral meglumine diatrizoate esophagogram during radiotherapy for esophageal cancer.

This trial was a prospective, multicenter, diagnostic clinical trial. First, we estimated the sample size using the test sample size estimation formula: n = uα 2po(1−po)/(p−po)2. Patients were selected according to the inclusion criteria of using radical radiotherapy to treat esophageal cancer. Weekly oral meglumine diatrizoate esophagogram was performed to screen for esophageal fistula during radiotherapy. The patient swallowed approximately 60 mL of 76% meglumine diatrizoate and underwent a radiographic examination; medical personnel observed the esophageal lesions to determine whether an esophageal fistula formed. If an esophageal fistula was observed, esophagofiberoscopy and computer tomography was used to further confirm the diagnosis; simultaneously, radiotherapy was stopped, and the patient received high intravenous nutrition and antiinflammatory therapies. The research team reviewed the esophagogram weekly to assess the recovery of the esophageal fistula; if the fistula healed, the patient continued to complete the radiotherapy program. Any serious adverse drug reactions were reported promptly to the hospital ethics committee. The diagnostic pathway shows in the Fig. 1.

For this study, 105 patients were recruited from 3 hospitals in Sichuan Province. There were no randomized control groups in this study. All patients who met inclusion criteria and provided informed consent could participate in the study.

#### 3.1.1 Inclusion criteria

(1) Pathologically proven, inoperable, initial diagnosis of esophageal cancer at stages I–IV.
(2) Esophageal lesions received radiotherapy.
(3) Eastern Cooperative Oncology Group physical status score was 0 to 1.
(4) Nonsurgical treatment of esophageal cancer at a standard clinical stage of I–IV.
(5) No esophageal perforation and active esophageal bleeding, no obvious trachea, thoracic major vascular invasion.
(6) Patient or family members signed the formal informed consent.

#### 3.1.2 Exclusion criteria

(1) Iodine allergy and hepatic and renal dysfunction.
(2) Active diagnoses of tuberculosis, multiple spinal tumors, hyperthyroidism.
(3) Patients who fail to understand the requirements of the trial or may not comply with the test requirements.
(4) There were obvious esophageal ulcers, moderate chest or back pain, or symptoms of esophageal perforation.

### 3.2 Data collection and management

We collected basic data for enrolled patients, with name, gender, age, lesion location, pathological type, clinical stage, radiotherapy dose, and frequency, examination results of weekly oral meglumine diatrizoate esophagogram. We reviewed and analyzed the data. For the statistical portion of this study, we used the data acquisition system electronic data management system to construct the database and capture and store all data. The main researchers performed statistical analysis prior to data audit. We performed data audit after data were locked according to the final statistical analysis plan.

### 3.3 Sample size calculation

With this method, we estimated that the sensitivity for esophageal fistula diagnosis is 45.5%, and the specificity is 97.8%. The sample size was calculated to be at least 105 cases. Our study was planned for 1-year period with 1-year follow-up. Patients received radiotherapy 5 times a week, for a total of 6 to 7 weeks of radiotherapy.

### 3.4 Statistical analysis and assessment of the primary and secondary endpoints

Diagnostic research evaluation shows in Table 1. In which a + b + c + d = 105, sensitivity = a/a + c × 100%, specificity = d/b + d × 100%, rate of missed diagnosis = 1, sensitivity = c/a + c × 100%. Healing rate of esophageal fistula = (number of cases of healing of esophageal fistula/number of cases of esophageal fistula) × 100%.

**Table 1:**
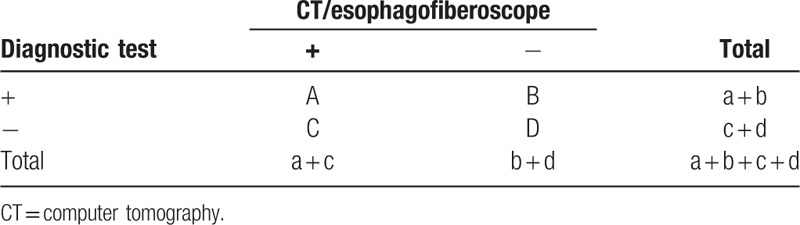
Diagnostic research evaluation form.

### 3.5 Stop research standards

Patients may withdraw from the study at any time for the following reasons:

(1) Patient withdrawal of informed consent
(2) Owing to patient safety events, researchers stop test
(3) When adverse events occur, researchers or patients opt to discontinue research
(4) Researchers exercise discretion

### 3.6 Ethics

The trial received ethical approval from the Ethics Committee of Mianyang Central Hospital, Sichuan, China (Number: S2017054). The entire experiment is subject to the supervision and management of the ethics committee.

### 3.7 Status

This study opened to recruitment in September 2017, with a planned recruitment period of 2 years.

## 4 Discussion

Esophageal fistula is a serious and common complication of radiation therapy, and is associated with high rates of morbidity and mortality, early diagnosis and treatment is important. But patients with early esophageal fistula exhibit no symptoms, they do not undergo screening tests until large fistulae have formed. The screening of esophageal fistula is necessary during radiotherapy. Oral meglumine diatrizoate esophagograms have many advantages including less sophisticated equipment requirements, low costs, simple operation, noninvasiveness, and fewer adverse reactions associated with the procedure. We designed this experiment to identify sensitivity and specificity of oral meglumine diatrizoate in an esophagogram for screening esophageal fistula during radiotherapy.

Expectations: Meglumine diatrizoate esophagogram can be used to screening esophageal fistula during radiotherapy, and more patients benefit from early detection and treatment of esophageal fistula.

## Author contributions

**Conceptualization:** Ying Ma, Xiaobo Du.

**Investigation:** He Hu, Yu Zhao, Lingli Fan, Zhenhua Zhao, Dongbiao Liao, Musheng Li, Miao Xiang.

**Project administration:** Rong Wu, Musheng Li.

**Resources:** Dongbiao Liao.

**Supervision:** Ying Ma.

**Writing – original draft:** Lidan Geng.

**Writing – review & editing:** Xiaobo Du.
